# Mortality Rate and Characteristics of Deaths Following COVID-19 Vaccination

**DOI:** 10.3389/fmed.2021.670370

**Published:** 2021-05-14

**Authors:** Gang Lv, Jing Yuan, Xiaomo Xiong, Minghui Li

**Affiliations:** ^1^Department of General Surgery, Chinese PLA General Hospital, Beijing, China; ^2^Department of Clinical Pharmacy and Pharmacy Administration, School of Pharmacy, Fudan University, Shanghai, China; ^3^Department of Clinical Pharmacy and Outcomes Sciences, College of Pharmacy, University of South Carolina, Columbia, SC, United States; ^4^Department of Clinical Pharmacy and Translational Science, University of Tennessee Health Science Center, Memphis, TN, United States

**Keywords:** COVID-19, vaccine, pharmacovigilance, safety, long-term care facility

## Abstract

**Background:** The emergency use authorization for coronavirus disease 2019 (COVID-19) vaccines brought both hopes and concerns to the Americans and others. We aimed to estimate the mortality rate of COVID-19 vaccination and presented characteristics of deaths following COVID-19 vaccination.

**Methods:** Data on deaths following COVID-19 vaccination were obtained from the Vaccine Adverse Event Reporting System (VAERS) from December 11, 2020 through January 8, 2021. The Centers for Disease Control and Prevention (CDC) COVID Data Tracker was used to identify the total number of people receiving COVID-19 vaccines during the same period to estimate the mortality rate. Stratified analysis was conducted by the location of vaccination.

**Results:** As of January 8, 2021, 55 deaths were reported, and the mortality rate of COVID-19 vaccination was 8.2 per million population. A total of 37 deaths were reported among long-term care facility residents, and the mortality rate was 53.4 per million population. Top reported comorbidities associated with deaths included hypertension, dementia, chronic obstructive pulmonary disease (COPD), diabetes, and heart failure. In addition, dementia was more likely to be associated with deaths vaccinated at long-term care facilities than at other locations.

**Conclusion:** The benefits of COVID-19 vaccines outweigh the potential risks in older frail populations, and our findings do not support actions to exclude older adults from being vaccinated. However, continued monitoring of COVID-19 vaccination is still warranted.

## Background

The emergency use authorization for coronavirus disease 2019 (COVID-19) vaccines brought both hopes and concerns to the Americans and others. Most recently, the Norwegian Medicines Agency reported 29 deaths following COVID-19 vaccines in very frail elderly patients, raising safety concerns for vaccination in older adults (1). Norwegian government has adjusted advice on who should receive COVID-19 vaccines which are based on novel messenger RNA (mRNA) technology.

In contrast, the U.S. has prioritized people aged 65 years and older and long-term care facility residents to receive COVID-19 vaccines. However, COVID-19 vaccines have not been tested in clinical trials in these frail older adults (2). Pharmacovigilance examines adverse events of drugs or vaccines in populations but this information on COVID-19 vaccines is limited. This study estimated the mortality rate of COVID-19 vaccination and presented characteristics of deaths following COVID-19 vaccination and top reported comorbidities and medications associated with deaths in the U.S.

## Methods

Data on deaths following COVID-19 vaccination were obtained from the Vaccine Adverse Event Reporting System (VAERS), a national vaccine safety surveillance program (3). We searched VAERS for deaths associated with any COVID-19 vaccine from December 11, 2020 through January 8, 2021. To solve the common limitation of passive surveillance data of unknown denominator, we used the Centers for Disease Control and Prevention (CDC) COVID Data Tracker for the total number of people receiving COVID-19 vaccines during the same period to estimate the mortality rate (4). Stratified analysis was conducted by the location of vaccination.

## Results

Among 6,688,231 individuals who received COVID-19 vaccination, 55 deaths were reported as of January 8, 2021. The mortality rate of COVID-19 vaccination was 8.2 per million population (Table 1). A total of 37 deaths were reported in long-term care facilities when 693,246 residents are vaccinated. The COVID-19 vaccination mortality rate was 53.4 per million population among long-term care facility residents.

**Table 1:** Characteristics of deaths following COVID-19 vaccination in the U.S. (*N* = 55).

	**All locations** ***N*** **=** **55**		**Long-term care facilities** ***N*** **=** **37**		**Other locations** ***N*** **=** **18**		***P***
	***N***	**%**	***N***	**%**	***N***	**%**	
**Age**							0.0003
18–64	9	16.4	1	2.7	8	44.4	
65–74	9	16.4	5	13.5	4	22.2	
75–84	12	21.8	9	24.3	3	16.7	
85+	25	45.5	22	59.5	3	16.7	
**Sex**							0.0444
Female	29	52.7	23	62.2	6	33.3	
Male	26	47.3	14	37.8	12	66.7	
**Region**							0.5464
Northeast	5	9.1	3	8.1	2	11.1	
Midwest	21	38.2	15	40.5	6	33.3	
South	17	30.9	13	35.1	4	22.2	
West	9	16.4	5	13.5	4	22.2	
Unknown	3	5.5	1	2.7	2	11.1	
**Days to death**							0.6989
0	14	25.5	11	29.7	3	16.7	
1–2	15	27.3	9	24.3	6	33.3	
3–6	16	29.1	11	29.7	5	27.8	
7+	10	18.2	6	16.2	4	22.2	

Adults aged 85 years and over accounted for half of the reported deaths (*N* = 25, 45.5%). Those vaccinated at long-term care facilities were more likely to be older and females than at other locations. A total of 14 individuals (25.5%) died on the same day and 45 individuals (81.8%) died within 1 week following vaccination. Top reported comorbidities associated with deaths were hypertension, dementia, chronic obstructive pulmonary disease (COPD), diabetes, and heart failure (Table 2). Dementia was more likely to be associated with deaths at long-term care facilities. Top medications associated with deaths were pain relievers, fever reducers, and antihypertensives.

**Table 2:** Top reported comorbidities and medications associated with deaths following COVID-19 vaccination by the location of vaccination.

	**All locations** ***N*** **=** **55**		**Long-term care facilities** ***N*** **=** **37**		**Other locations** ***N*** **=** **18**		***P***
	***N***	**%**	***N***	**%**	***N***	**%**	
**Comorbidities**							
Hypertension	22	40.0	14	37.8	8	44.4	0.6390
Dementia	17	30.9	15	40.5	2	11.1	0.0267
COPD	13	23.6	9	24.3	4	22.2	1.0000
Diabetes	12	21.8	7	18.9	5	27.8	0.4993
Heart failure	10	18.2	9	24.3	1	5.6	0.1399
Hyperlipidemia	9	16.4	6	16.2	3	16.7	1.0000
Anemia	8	14.5	7	18.9	1	5.6	0.2497
CKD	7	12.7	4	10.8	3	16.7	0.6713
Stroke	7	12.7	5	13.5	2	11.1	1.0000
Atrial fibrillation	6	10.9	5	13.5	1	5.6	0.6514
Depression	6	10.9	5	13.5	1	5.6	0.6514
Fall	6	10.9	6	16.2	0	0.0	0.1621
GERD	6	10.9	4	10.8	2	11.1	1.0000
**Medications**							
Acetaminophen	9	16.4	8	21.6	1	5.6	0.2441
Aspirin	9	16.4	6	16.2	3	16.7	1.0000
Amlodipine	8	14.5	6	16.2	2	11.1	0.7079
Metoprolol	8	14.5	6	16.2	2	11.1	0.7079
Omeprazole	7	12.7	6	16.2	1	5.6	0.4058
Potassium	6	10.9	6	16.2	0	0.0	0.1621
Vitamin D	6	10.9	6	16.2	0	0.0	0.1621
Docusate	5	9.1	5	13.5	0	0.0	0.1603
Furosemide	5	9.1	5	13.5	0	0.0	0.1603
Lisinopril	5	9.1	4	10.8	1	5.6	0.6583
Mirtazapine	5	9.1	4	10.8	1	5.6	0.6583
Tamsulosin	5	9.1	3	8.1	2	11.1	1.0000

*COVID-19, coronavirus disease 2019; COPD, chronic obstructive pulmonary disease; CKD, chronic kidney disease; GERD, gastroesophageal reflux disease*.

## Discussion

COVID-19 vaccine is safe in younger groups. The majority of the reported deaths were in people aged 85 and older and vaccinated at long-term care facilities; these patients are frail older people with serious underlying health conditions such as dementia, hypertension, heart failure, COPD, diabetes, anemia, and fall. In addition, these vulnerable patients are polypharmacy users. Certain vaccine-disease and vaccine-drug interactions might have contributed to or have worsened health outcomes of those already vulnerable populations. It is essential to monitor the allergic reactions following the vaccination that mainly occur within a short period of time for preventable risks (5). However, the mortality rate of 53.4 per million following COVID-19 vaccination among long-term care facility residents during the study period was much lower compared to the 2019 monthly all-cause mortality rate of 0.3% among adults aged 65 years or older (6), or the 30-day all-cause mortality rate of 21.5% among US nursing home residents with COVID-19 (7). Therefore, our data suggest that the benefits of COVID-19 vaccines far outweigh the potential risks in older frail populations (e.g., long-term care facilities), and our findings do not support actions to exclude older adults from being vaccinated as the Norwegian government did. Continued monitoring of COVID-19 vaccination among the older population, particularly those with comorbidities and medications reported in this study, however, is warranted.

## Data Availability Statement

Publicly available datasets were analyzed in this study. This data can be found at: https://vaers.hhs.gov/.

## Ethics Statement

The studies involving human participants were reviewed and approved by the University of Tennessee Health Science Center. Written informed consent for participation was not required for this study in accordance with the national legislation and the institutional requirements.

## Author Contributions

GL and ML: concept and design. JY and ML: acquisition, analysis, or interpretation of data. GL, JY, XX, and ML: drafting of the manuscript and critical revision of the manuscript for important intellectual content. GL, JY, and ML: statistical analysis. All authors contributed to the article and approved the submitted version.

## Conflict of Interest

The authors declare that the research was conducted in the absence of any commercial or financial relationships that could be construed as a potential conflict of interest.
